# Circulating ESR1, long non-coding RNA HOTAIR and microRNA-130a gene expression as biomarkers for breast cancer stage and metastasis

**DOI:** 10.1038/s41598-023-50007-5

**Published:** 2023-12-19

**Authors:** Noura R. Abdel-hamid, Eman A. Mohammed, Eman A. Toraih, Mahmoud M. Kamel, Ahmed Samir Abdelhafiz, Fouad M. Badr

**Affiliations:** 1 Genetics Unit, Department of Histology and Cell Biology, Faculty of Medicine, Suez Canal University, Ismailia, Egypt; 2 Division of Endocrine and Oncologic Surgery, Department of Surgery, School of Medicine, Tulane University, New Orleans, LA 70112 USA; 3 Department of Clinical Pathology, National Cancer Institute, Cairo University, Kasr Al-Aini Street, From El-Khalig Square, Cairo, 11796 Egypt; 4 Baheya Centre for Early Detection and Treatment of Breast Cancer, Giza, Egypt

**Keywords:** Cancer, Diagnostic markers, Predictive markers

## Abstract

Breast cancer, the most prevalent cancer among women, has posed a significant challenge in identifying biomarkers for early diagnosis and prognosis. This study aimed to elucidate the gene expression profile of Estrogen Receptor-1 (ESR-1), long non-coding RNA HOTAIR, and microRNA-130a in the serum of Egyptian breast cancer patients, evaluating the potential of HOTAIR and miR-130a as biomarkers for predicting pathological parameters in BC. The study involved 45 patients with primary BC, with serum samples collected preoperatively and postoperatively twice. The expression levels of ESR-1, HOTAIR, and miR-130a were quantified using real-time PCR and analyzed for correlations with each other and with the clinical and pathological parameters of the patients. Serum HOTAIR levels exhibited a strong positive association with metastasis and demonstrated a significant increase after 6 months in all patients with locally advanced and stage IV BC. Conversely, tumors with advanced stages and metastatic lesions showed significantly lower expression levels of miR-130a. Notably, a significant positive correlation was observed between preoperative ESR-1 expression and both HOTAIR and miR-130a levels. Serum HOTAIR and miR-130a levels have emerged as promising non-invasive biomarkers with the potential to predict the pathological features of BC patients. HOTAIR, an oncogenic long non-coding RNA (lncRNA), and miR-130a, a tumor suppressor miRNA, play crucial roles in tumor progression. Further investigations are warranted to elucidate the intricate interplay between HOTAIR and miR-130a and to fully comprehend the contribution of HOTAIR to BC recurrence and its potential utility in early relapse prediction.

## Introduction

Breast cancer (BC) stands as the most prevalent and fatal cancer among women worldwide^1,2^. Egypt mirrors this global trend, with breast cancer holding the top position among female cancers in the country^3^. This formidable disease exhibits remarkable heterogeneity at both phenotypic and genetic levels, characterized by a high degree of variation in both domains^4^. The underlying mechanisms driving this heterogeneity stem from differences at the genomic, epigenomic, transcriptomic, and proteomic levels. These intricate variations translate into a wide spectrum of tumor cell behaviors, ranging from growth rate and aggressiveness to treatment response^5^. The remarkable heterogeneity of breast cancer poses a significant challenge in identifying an ideal biomarker for early diagnosis and prognosis. This is due to the limitations of individual biomarkers in accurately detecting all breast cancers or predicting their clinical progression^6^.

Diverse classes of RNA molecules, including long non-coding RNAs (lncRNAs) and microRNAs (miRNAs), have emerged as key regulatory elements in various diseases, including cancer. LncRNA HOTAIR, one of the first lncRNAs associated with cancer, has garnered significant attention due to its involvement in the development and progression of various malignancies^7,8^. Its potential as a diagnostic and therapeutic biomarker across different cancer types is currently under investigation^9^. Similarly, microRNA-130a has been implicated in the pathogenesis of various gynecological malignancies, including cervical and ovarian cancer^10^. A compelling interplay between HOTAIR and miR-130a has been observed in certain cancer types, including gallbladder cancer, where it promotes tumor invasion and metastasis^11^.

Estrogen receptor (ER) expression plays a pivotal role in breast cancer development^12^, with approximately 70% of BC patients exhibiting elevated ER gene expression levels^13^. ER activates a vast network of genes that promote breast cancer cell proliferation and survival^14^. Intriguingly, studies have demonstrated that ER, in conjunction with its regulatory proteins, can directly interact with the HOTAIR promoter region, thereby modulating its expression in cells^15^.

This study aims to quantify the expression levels of the ESR1, HOTAIR, and miR-130a genes in serum samples obtained from Egyptian female breast cancer patients and examine the correlations between their expression levels and the clinicopathological features of the disease. Additionally, the study seeks to evaluate the potential of HOTAIR and miR-130a as non-invasive biomarkers for predicting pathological parameters in breast cancer.

## Patients and methods

### Study participants

The study cohort comprised 45 adult female patients with primary breast cancer, confirmed by a combination of clinical, radiological, and pathological assessments. These patients had not received neoadjuvant chemotherapy or radiotherapy prior to surgery. An equal number of healthy age-matched controls were selected based on age, gender, timing of sample collection, and duration of specimen storage. Patients with a history of chemotherapy or radiotherapy, secondary breast cancer (metastatic from any primary site), any other malignancies at the time of diagnosis, or conditions known to alter microRNA expression, such as pregnancy or lactation within the past year, heart disease, renal problems, or diabetes mellitus, were excluded from the study.

Between May and December 2022, the patients were recruited from the outpatient clinic at Suez Canal University Hospitals in Egypt. The study received ethical approval from the Medical Research Ethics Committee of the Faculty of Medicine, Suez Canal University, and all participants provided written informed consent.

Following surgical excision, breast tumor specimens were subjected to pathological evaluation to determine tumor type, size, pathological grade (as per the Elston and Ellis modification of the Scarff–Bloom–Richardson classification)^16,17^, and lymph node involvement. The American Joint Committee on Cancer (AJCC) tumor-node-metastasis (TNM) classification system was employed to determine the clinical stage at the time of surgery^18^. Prognostic assessment was performed using the Nottingham Prognostic Index (NPI) and the Immunohistochemical Prognostic Index (IHPI)^19,20^. All procedures were conducted in adherence to relevant guidelines and regulations. Patients were monitored for 6 months following surgery, with clinical data and investigations being collected throughout the follow-up period.

### Methods

Serum blood samples were collected preoperatively just before mastectomy and postoperatively twice (2 weeks and 6 months after surgery) to assess changes in marker levels compared to preoperative levels. RNA extraction from the serum was performed using the Qiagen miRNeasy Mini kit (Qiagen, Cat. No. 217004) following the manufacturer's instructions. Quantitative real-time PCR (RT-qPCR) was employed using a two-step approach: Quantitative real-time PCR (RT-qPCR) was employed using a two-step approach:

1. Reverse Transcription (RT): Two kits were used for reverse transcription:
  - TaqMan^®^ High-Capacity RNA-to-cDNA Master mix (Applied Biosystems, Part No. 4390777) and specific primers (ESR1 5× and HOTAIR 5× (Applied Biosystems, Cat. No. 4331182), along with TaqMan^®^ GAPDH control reagent (human) for Glyceraldehyde-3-Phosphate Dehydrogenase (GAPDH) (Applied Biosystems, Cat. No. 402869), labelled with VIC dye as a reporter. GAPDH was used to normalize the expression levels of ESR1 and HOTAIR genes, correcting for any potential variations in RNA quantity or quality across samples. The tube was stored at − 200 °C and protected from light.
  - TaqMan^®^ MicroRNA reverse transcriptase kit (Applied Biosystems, Cat. No. 4366596) and specific microRNA primers (hsa-miR-130a 5x (Applied Biosystems, Cat. No. 4427975) and TBP 5× (Applied Biosystems, Cat. No. 4331182). Human TBP was used as an internal control.
2. Real-time PCR:
  - TaqMan^®^ Gene Expression Master Mix (Applied Biosystems, Cat. No. 4369016) and TaqMan^®^ assays (for hsa-miR-130a 20× and TBP 20×) were used for real-time PCR.
  - For ESR1, HOTAIR, and GAPDH, qPCR GreenMaster Mix (Jena Bioscience, Cat. No. PCR-336S) and specific primers (Sangon Biotech Co., Ltd., Shanghai, China) were employed.

The sequences of the primers used in this study are listed in Supplementary Table 1, and the thermal cycling conditions are provided in Supplementary Tables 2 and 3.

Real-time PCR was conducted using an AB 7500HT instrument equipped with SDS Software version 2.1.1 (Applied Biosystems, Egypt). Relative quantification was employed for data analysis, and results were presented using the ΔΔCT method^14^.

### Statistical analysis

Data management was performed using the Statistical Package for the Social Sciences (SPSS) for Windows software, version 22.0. Descriptive statistics were presented as median (quartiles) for quantitative variables and percentages for qualitative variables. The Chi-square test was employed for statistical analysis. Due to the non-Gaussian distribution of HOTAIR and miR-130a relative expression levels, their levels were characterized using median values and ranges encompassing the 25th and 75th percentiles. The expression of HOTAIR and miR-130a across different groups was analyzed using non-parametric tests: the Friedman test was applied for paired samples, the Mann–Whitney *U* test for comparisons involving two independent groups, and the Kruskal–Wallis test for comparisons involving three or more independent groups. Correlation analysis was performed using Spearman's rank correlation coefficient (rs). Receiver Operating Characteristic (ROC) curves were constructed to evaluate the optimal cut-off values for case–control status prediction, maximizing sensitivity and specificity. Kaplan–Meier survival curves were employed to determine overall survival, followed by the log-rank test to assess the statistical significance of differential survival rates. All statistical tests were two-tailed, and a probability value (*P*) below 0.05 was considered statistically significant^15^.

## Results

### General characteristics of the study groups

The majority of patients (55.5%) fell within the age range of 40 to 60 years, with a median age of 57 (range 46–62). Approximately half of the patients were premenopausal. Invasive ductal carcinoma was the most prevalent histological type identified in tissue samples (66.7%), followed by invasive lobular carcinoma (11.1%). Based on the Scarff–Bloom–Richardson classification, 11.1%, 55.6%, and 33.3% of carcinomas were categorized as pathological grades I, II, and III, respectively. At the time of diagnosis, 64.4% of patients (29 patients) exhibited axillary nodal involvement. Only 4 patients (4.4%) presented with metastases, with two cases involving bone metastases and two involving both bone and liver metastases.

In terms of receptor status, most patients (34, 75.6%) were diagnosed with luminal A breast cancer, while 4 patients (8.8%) and 5 patients (11.1%) were classified as luminal B and basal-like subtypes, respectively. Additional clinical and pathological data for the patients are presented in Table 1, Supplementary Tables 4 and 5, and Supplementary Figs. 1 and 2.

**Table 1:** Clinicopathological data of breast cancer patients (n = 45).

Variables	Number	Percentage
Side		
Right	22	48.9
Left	20	44.4
Bilateral	3	6.7
*Site		
UOQ	18	40
UIQ	3	6.7
LOQ	4	8.8
LIQ	4	8.8
Retroareolar	4	8.8
Axillary tail	4	8.8
≥ 2 sides	8	17.8
Histological type		
Infiltrative ductal carcinoma	30	66.7
Infiltrative lobular carcinoma	5	11.1
Mixed ductal and lobular	3	6.7
Micropapillary carcinoma	2	4.4
Invasive cribriform carcinoma	2	4.4
Medullary carcinoma	1	2.2
Mucinous carcinoma	1	2.2
Adenocarcinoma	1	2.2
Pathological grade		
Grade 1	5	11.1
Grade 2	25	55.6
Grade 3	15	33.3
Tumor size		
T1	14	31.1
T2	17	37.8
T3	7	15.6
T4	7	15.6
Lymph node status		
N0	16	35.6
N1	25	55.5
N2	3	6.7
N3	1	2.2
Lympho-vascular invasion	15	33.3
Distant metastasis	4	8.8
Bone and liver	2	4.4
Bone only	2	4.4
Clinical stage		
Stage I	8	17.8
Stage II A	10	22.2
Stage II B	3	6.6
Stage III A	6	13.3
Stage III B	10	22.2
Stage III C	4	8.8
Stage IV	4	8.8
Nottingham Prognostic Index		
Good prognosis	7	15.6
Moderate prognosis	21	46.7
Poor prognosis	17	37.8
Immunohistochemical Prognostic Index		
Good prognosis (0)	27	60
Good prognosis (1)	10	22.2
Moderate prognosis (2)	6	13.3
Poor prognosis (3)	2	4.4

*Site: *UOQ* upper outer quadrant, *UIQ* upper inner quadrant, *LIQ* lower inner quadrant, *LOQ* lower outer quadrant.

### Molecular results

#### Association between preoperative ESR1 expression and clinico-pathological features in BC patients

Tumors with the luminal B molecular subtype exhibited higher levels of ESR1 expression compared to other molecular subtypes, although this difference did not reach statistical significance. ESR1 expression levels were significantly associated with higher tumor grade (*P* = 0.003), as shown in Supplementary Fig. 3. While no significant association was found between ESR1 levels and tumor size, a notable difference in fold change was observed.

Tumors with advanced clinical stage had significantly higher levels of ESR1 (*P* = 0.032), as illustrated in Supplementary Fig. 4. Additionally, higher levels of ESR1 were observed in patients with poor prognosis for NPI (*P* = 0.046), as depicted in Supplementary Fig. 5. However, no significant associations were found between ESR1 levels and tumor side, location, metastasis, or IHPI.

#### Association between preoperative HOTAIR expression and clinico-pathological features in BC patients

Tumors of the luminal B molecular subtype and those with estrogen receptor positivity exhibited higher levels of HOTAIR expression compared to other molecular subtypes, although these differences did not reach statistical significance. Tumors of high grade were also associated with elevated HOTAIR expression levels.

HOTAIR expression levels were associated with tumors larger than 5 cm in size, but this association did not reach statistical significance. Tumors with advanced clinical stage showed a trend towards higher HOTAIR levels, but this difference was not statistically significant (*P* = 0.077). No significant associations were found between HOTAIR levels and tumor side, location, metastasis, or NPI index.

#### Correlation between HOTAIR expression levels pre-operatively, post-operatively and after 6 months

**Figure 1:**
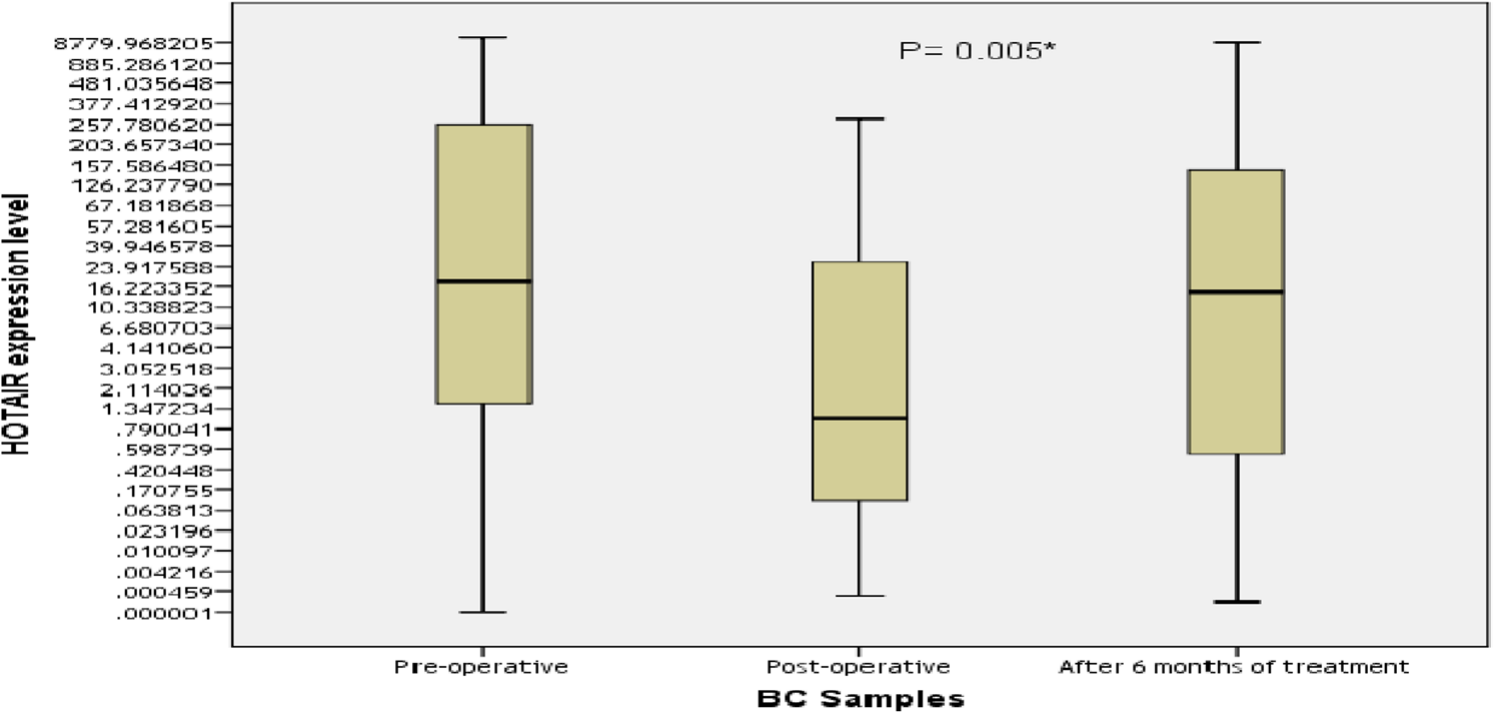
Association between HOTAIR expression levels and different BC patients. Samples (n = 45). Values are represented as medians. The box defines upper and lower quartiles (25% and 75%, respectively) and the error bars indicate upper and lower adjacent limits. Friedman test was used.

The mean CT values for HOTAIR expression differed significantly between breast cancer patients and healthy controls (*P* = 0.04), indicating elevated levels of HOTAIR in patients. The changes in HOTAIR expression before and after surgery are detailed in Table 2. A significant difference was observed in HOTAIR expression levels among breast cancer patients preoperatively, postoperatively, and after 6 months (Spearman's rho = 0.114, *P* = 0.005), as illustrated in Fig. 1.

**Table 2:** HOTAIR expression levels in breast cancer patients (n = 45). Significant values are in bold.

Variables	Control	BC			P value
		Pre-operative	Post-operative	After 6 months	
Mean C_T_					
GAPDH	30 (29.2–30.4)	32.3 (29.3–37.7)	30.3 (28.8–31.5)	30.5 (28.5–31.4)	0.170
HOTAIR	31.5 (29.5–35.8)	30.7 (31.4–35.1)	32.7 (28–36.8)	27.9 (24.3–37.1)	0.04*
∆ C_T_	2.1 (− 0.3 to 6.1)	− 1.12 (− 6.73 to 3.5)	2.09 (− 2.21 to 5.9)	− 2.5 (− 5 to 8.7)	0.01*
∆∆ C_T_		− 4.1 (− 8.1 to − 0.5)	0.13 (− 4.9 to 2.9)	− 0.39 (− 7.4 to 1.9)	0.000*
Fold change		17.15 (1.41–282.2)	0.91 (0.13–32.3)	16.64 (0.41–177.5)	0.005*

Values are shown as median (1st–3rd quartiles). Kruskal–Wallis and Friedman tests were used. CT; threshold cycle number. ∆∆CT and fold change calculated versus control. *Significantly different at P value < 0.05.

**Figure 2:**
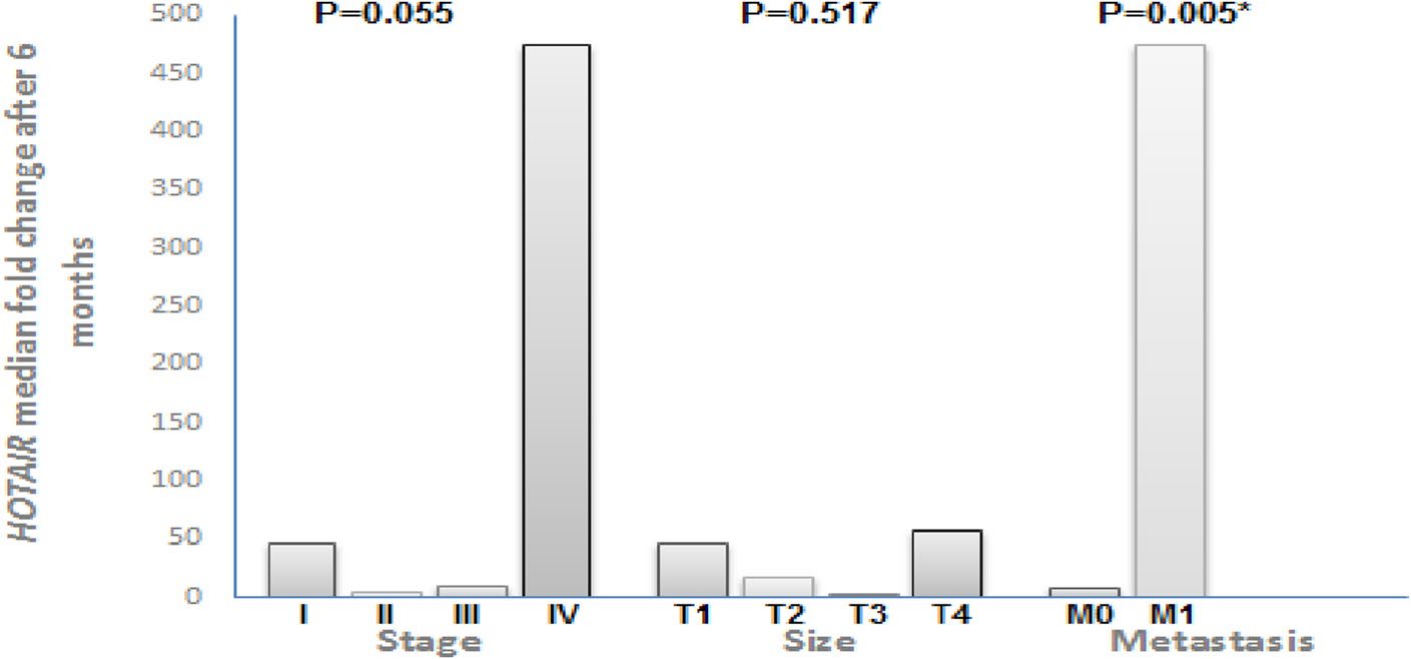
Association between HOTAIR expression after 6 months of treatment in BC patients with tumor size, stage, and metastasis (n = 26).

Notably, a significant increase in HOTAIR expression was observed after 6 months of surgery in all patients who developed metastasis, suggesting a potential association between HOTAIR levels and disease progression. This was further supported by the strong positive correlation observed between serum HOTAIR expression and the occurrence of metastasis (Spearman's rho = 0.526, *P* = 0.005). Additionally, a significant increase in HOTAIR expression was detected after 6 months in all patients with locally advanced breast cancer and stage IV breast cancer, as shown in Fig. 2.

#### MiR-130a expression profile and association with disease characteristics

**Figure 3:**
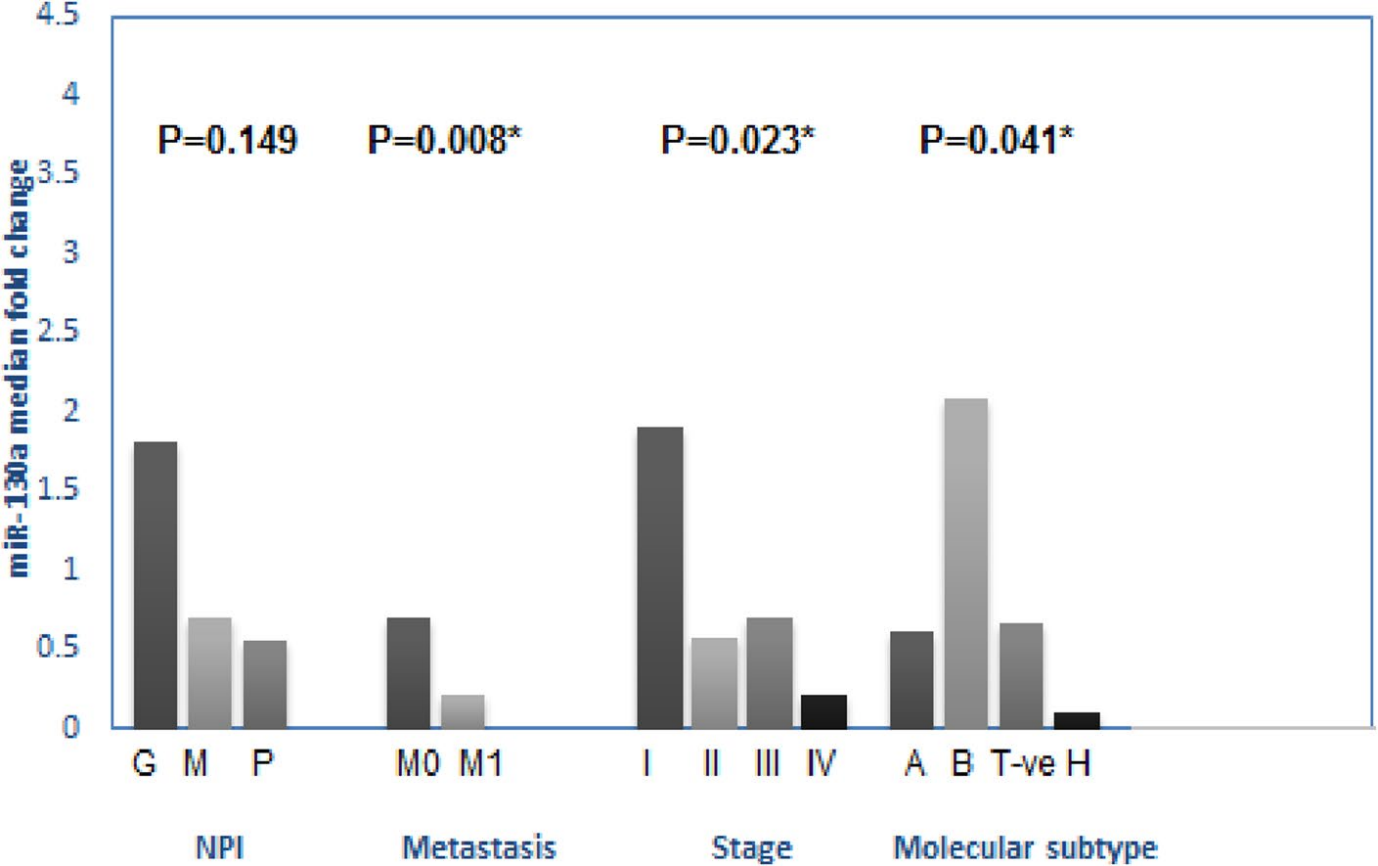
Association between miR-130a expression in BC patients and clinico-pathological characteristics (molecular subtypes: A, for luminal A; B, for luminal B; T−ve, for triple negative, and H, for Her + 2), stage, metastasis and NPI (Nottingham Prognostic Index; G, Good; M, Moderate and P, Poor).

Significant differences in mean CT values were observed for microRNA-130a between cancer patients and healthy controls (*P* = 0.0001), indicating elevated levels of miR-130a in patients (Table 3). Tumors with the luminal B molecular subtype exhibited significantly higher levels of miR-130a expression compared to other molecular subtypes (*P* = 0.04). Interestingly, tumors with stage I breast cancer had the highest levels of miR-130a expression, while those with advanced stage (stage IV) had the lowest levels (*P* = 0.023). This suggests a potential association between miR-130a expression and disease progression. Furthermore, a significant inverse correlation was observed between miR-130a levels and metastasis, with metastatic cases exhibiting significantly lower expression (*P* = 0.008) (Fig. 3).

**Table 3:** miR-130a expression levels in breast cancer patients (n = 45). Significant values are in bold.

Variables	Control	BC			P value
		Pre-operative	Post-operative	After 6 months	
Mean C_T_					
TBP	32.27 (32.27–35.27)	34.43 (32.61–34.60)	30.3 (28.8–31.5)	30.5 (28.5–31.4)	0.244
miRNA-130a	31. 6 (28.37–34.79)	34.05 (31.43–36.89)	34.67 (32.21–37.87)	36.43 (31.06–42.16)	0.000*
∆ C_T_	2.16 (− 1.86 to 4.56)	2.71 (− 1.56 to 4.77)	3.2 (− 2.75 to 7.05)	6.51 (0.79–12.73)	0.01*
∆∆ C_T_		0.65 (− 0.54 to 1.19)	1.35 (− 1.65 to 3.54)	7.4 (0.05–9.89)	0.000*
Fold change		0.64 (0.44–1.49)	0.39 (0.08–3.14)	0.006 (0.001–1.09)	0.000*

Values are shown as median (1st–3rd quartiles). Kruskal–Wallis and Friedman tests were used. CT; threshold cycle number. ∆∆CT and fold change calculated versus control. *Significantly different at P value < 0.05.

#### Co-expression profile of ESR1, HOTAIR, and miR-130a in breast cancer patients

As shown in Table 4, an analysis of preoperative expression levels revealed a significant positive correlation between ESR1 and HOTAIR (Spearman's rho correlation coefficient (rs) = 0.544, *P* = 0.000), suggesting a potential co-regulatory relationship between these two genes. Similarly, a significant positive correlation was observed between ESR1 and miR-130a (Spearman's rho (rs) = 0.341, *P* = 0.022), indicating a potential link between ESR1 and miR-130a expression. However, the correlation between HOTAIR and miR-130a expression was not significant (Spearman's rho (rs) = 0.276, *P* = 0.067), suggesting a less direct relationship between these two genes.

**Table 4:** Correlation between ESR1, HOTAIR and miR-130a expression (preoperatively) in BC patients (n = 45).

Variables	Pre-operative		Post-operative		After 6 months	
	Spearman's rho (rs)	P value	Spearman's rho (rs)	P value	Spearman's rho (rs)	P value
ESR1 HOTAIR	0.544	0.000*	0.234	0.123	0.336	0.126
ESR1 MiR-130a	0.341	0. 022*	0.007	0.967	0.197	0.433
HOTAIR MiR-130a	0.276	0.067	− 0.091	0.560	0.005	0.982

Spearman rho: Spearman's rho correlation coefficient (rs). *Significantly correlated.

#### Potential use of HOTAIR and miR-130a as non-invasive biomarkers in breast cancer

**Figure 4:**
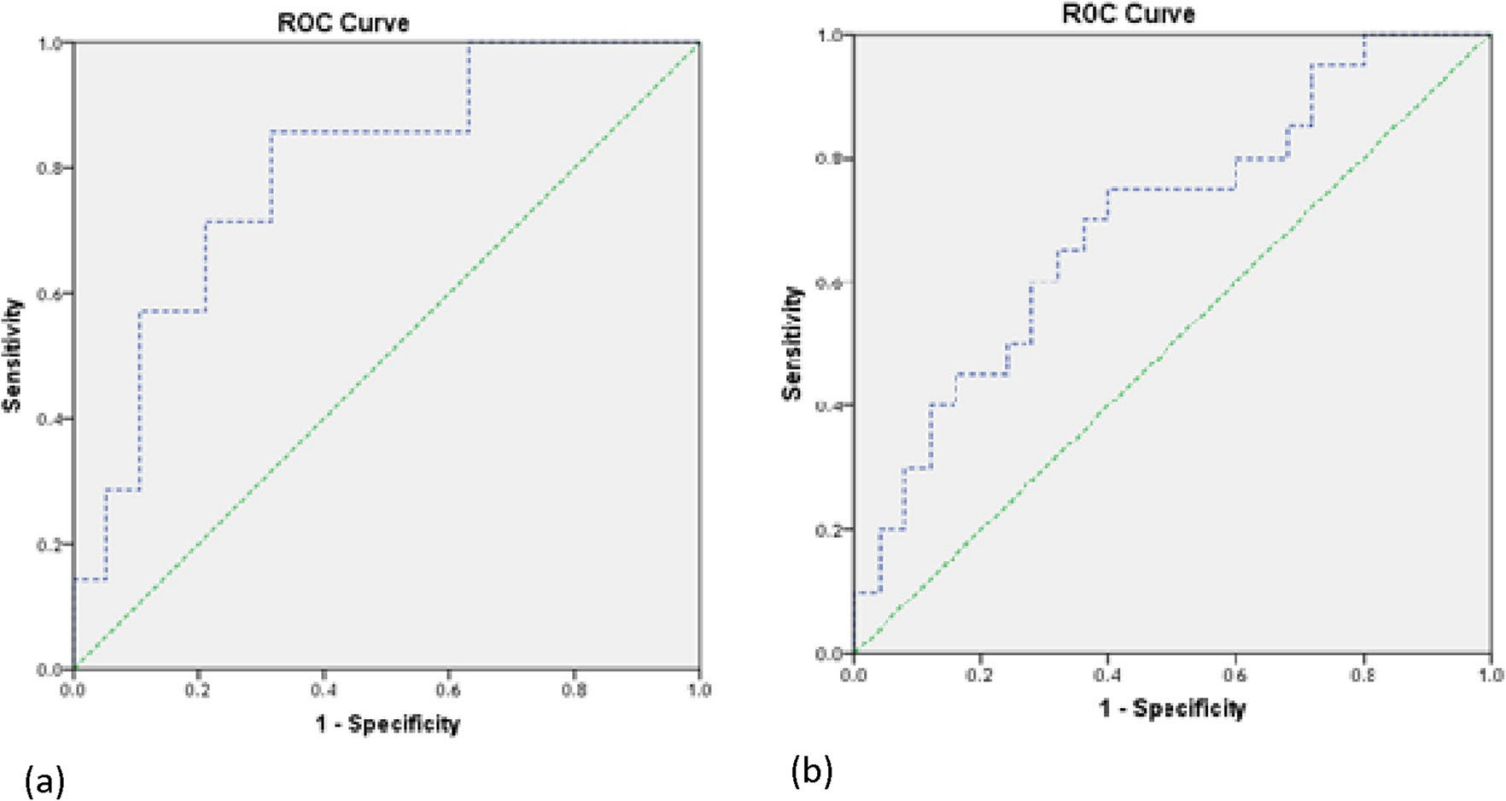
ROC curve of serum HOTAIR levels in BC patients regarding (**a**) tumor size and (**b**) stage of BC *ROC* receiver operating characteristic.

**Figure 5:**
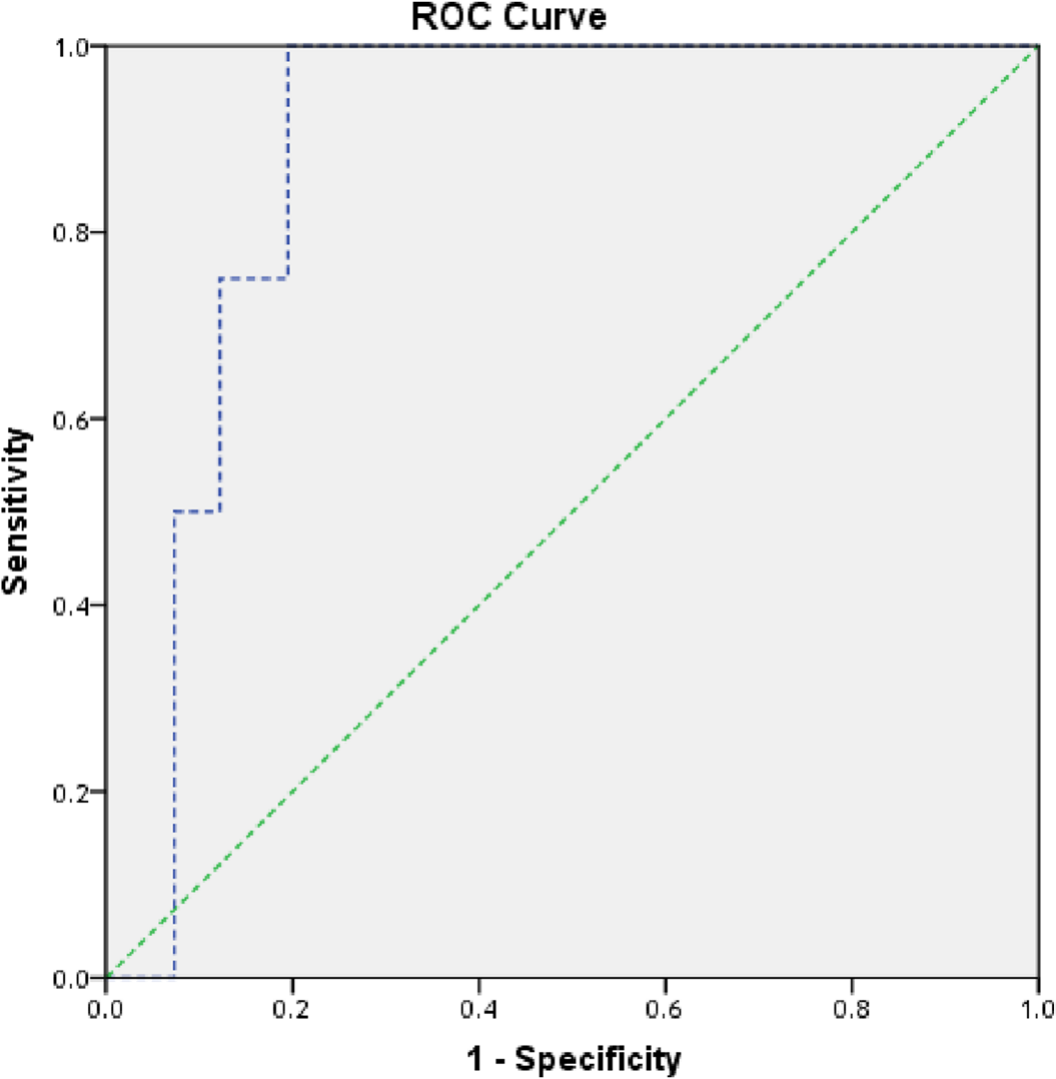
ROC curve of serum miR-130a levels in BC patients regarding stage and metastasis of BC. *ROC* receiver operating characteristic.

To evaluate the diagnostic potential of HOTAIR and miR-130a levels in serum samples, receiver operating characteristic (ROC) analysis was performed. Serum HOTAIR levels demonstrated a good ability to discriminate tumors larger than 5 cm from smaller tumors, with 86% sensitivity and 68% specificity (area under the curve [AUC] = 0.797 at the cutoff point of 95.79-fold). Additionally, serum HOTAIR levels effectively distinguished patients with stage III/IV breast cancer from those with earlier stages, exhibiting 75% sensitivity and 64% specificity (AUC = 0.698 at the cutoff point of 13.69-fold) (Fig. 4). Remarkably, serum miR-130a levels exhibited excellent ability to distinguish patients with metastasis and those with stage IV BC from patients with earlier stages of BC, with 100% sensitivity and 81% specificity (AUC = 0.884 at the cutoff point of 0.47-fold) (Fig. 5).

### ESR1 expression and survival

**Figure 6:**
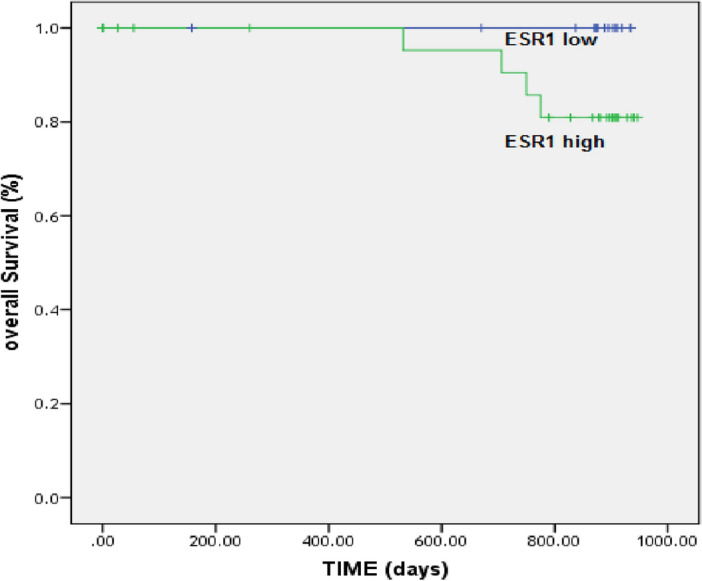
Kaplan–Meier plot for the association between serum ESR1 expression levels with patients’ survival in breast cancer.

To investigate the association between ESR1 expression and patient survival, survival analysis was conducted over a 2.5-year period. Kaplan–Meier analysis revealed a significantly shorter overall survival for patients with ESR1 overexpression (P < 0.05). Patients with high ESR1 expression exhibited a significantly shorter median survival time (1.5 years) compared to those with low ESR1 expression (3.5 years). Notably, all four patients who died during the study period had initially high levels of ESR1 expression (Fig. 6), suggesting a potential link between ESR1 overexpression and reduced survival in breast cancer patients.

## Discussion

Breast cancer is a global public health concern, representing a leading cause of morbidity and mortality among women worldwide. The development of novel biomarkers for screening, staging, and prognosis is crucial for effective prevention, patient stratification, personalized treatment, and improved management of the disease^21^. In this study, we demonstrate a significant correlation between the expression levels of ESR1, HOTAIR, and miR-130a in the serum of breast cancer patients. Furthermore, we observed associations between HOTAIR and miR-130a expression levels and several clinical characteristics of the disease, including tumor size, stage, and metastasis.

An elevation in HOTAIR expression was observed in the serum of breast cancer patients. Interestingly, HOTAIR expression levels underwent a transient decline postoperatively but rebounded after 6 months of treatment. This pattern mirrors the bimodal distribution of HOTAIR expression reported in primary breast cancer by Sørensen et al.^22^. Notably, a significant increase in HOTAIR expression was detected after 6 months of treatment in all patients with metastasis, as well as in all patients with locally advanced breast cancer and stage IV breast cancer. These findings suggest a potential role of HOTAIR as a prognostic marker and therapeutic target in breast cancer.

Our findings align with those of Lu et al., who demonstrated that HOTAIR expression could serve as a potential independent prognostic marker for primary breast cancer^23^. Several studies have indicated that elevated HOTAIR levels are a significant predictor of subsequent metastasis and poor prognosis^24–26^. HOTAIR plays a crucial role in maintaining breast cancer stem cells and is highly expressed in these cells derived from two cancer cell lines^27^. Cancer stem cells (CSCs) are a subset of cancer cells with the ability to self-renew and give rise to other cancer cells. They are believed to play a fundamental role in cancer recurrence, as they can survive treatment and generate new tumors. These findings suggest that HOTAIR may be a valuable biomarker for identifying patients at high risk of developing metastatic disease and for guiding treatment decisions. We propose long-term follow-up studies to assess the utility of HOTAIR in detecting breast cancer relapse.

Our findings revealed higher HOTAIR expression levels in tumors of the luminal B molecular subtype compared to other molecular subtypes and in patients with estrogen receptor positivity (ER+), although this difference did not reach statistical significance. Additionally, a significant positive correlation was observed between ESR1 and HOTAIR expression levels in breast cancer patients. These findings suggest that HOTAIR may serve as a potential prognostic biomarker for ER+ breast cancer due to its overexpression in this patient group^22^.

The HOTAIR gene promoter harbors binding sites for various transcription factors, including several estrogen response elements (EREs) that are targeted by the activated estrogen–estrogen receptor (ER) complex^15,28^. Previous studies have demonstrated that HOTAIR can function as an intermediary for ER signalling, even in malignant cells with low estrogen expression^29^. Collectively, our findings contribute to the growing body of evidence supporting a correlation between ESR1 and HOTAIR in breast cancer pathogenesis.

Our study revealed a significant downregulation of serum miR-130a in breast cancer patients preoperatively, post-operatively, and after 6 months of treatment. This pioneering investigation delved into the expression profile of miR-130a in the serum of breast cancer patients, elucidating its pre- and postoperative levels. Notably, we observed a significant association between miR-130a expression and breast cancer stage. Tumors with early-stage breast cancer (stage I) exhibited the highest miR-130a levels, while those with advanced-stage breast cancer (stage IV) demonstrated the lowest levels. Moreover, a substantial downregulation of this biomarker was evident in all metastatic cases.

Previous studies have revealed the dual nature of miR-130a in cancer, exhibiting both tumor suppressor and oncogenic properties depending on the cellular context^30–33^. Mallela et al.^31^ demonstrated the oncogenic role of miR-130a in oral squamous cell carcinoma, while Lohcharoenkal et al.^32^ suggested its tumor suppressor function in cutaneous squamous cell carcinoma. Boll et al. further underscored the oncogenic potential of miR-130a by highlighting its ability to repress the mitogen-activated protein kinase (MAPK) signaling pathway, thereby promoting vascular endothelial cell proliferation and angiogenesis in tumors^33^. These findings collectively suggest that the role of miR-130a in cancer is intricate and influenced by a multitude of factors, including the type and stage of cancer, as well as the patient's genetic background.

Two studies have shed light on the tumor suppressor role of miR-130a in breast cancer. Pan et al. demonstrated a direct interaction between miR-130a and RAB5A, a regulator of vesicle trafficking from the serum membrane to endosomes, which has been implicated in cancer metastasis^34^. Another study revealed that miR-130a inhibits breast cancer cell migration and invasion by targeting FOSL1, a transcription factor that regulates tumor cell proliferation and survival, suggesting that miR-130a may suppress the growth and metastasis of breast cancer cells^35^. These studies provide potential explanations for the tumor suppressor role of miR-130a observed in our study.

Our study revealed an association between HOTAIR and miR-130a expression levels, although statistical significance was not attained. Preoperatively, HOTAIR overexpression was linked to miR-130a downregulation. Conversely, postoperatively, both genes exhibited consistent downregulation. A negative correlation between HOTAIR and miRNA-130a has been observed in gallbladder cancer tissues^8^. Ma et al. identified a miRNA-130a binding site within HOTAIR, suggesting its crucial role in regulating miRNA-130a expression. Furthermore, HOTAIR's oncogenic activity is partly attributed to its negative regulation of miRNA-130a through the competitive endogenous RNA (ceRNA) network^36^. Wenxing et al. further elucidated the oncogenic potential of HOTAIR by demonstrating its ability to promote breast cancer cell growth and metastasis via the miR-130a-3p/Suv39H1 axis, mediated through the AKT/mTOR pathway. Overexpression of HOTAIR led to downregulation of miR-130a-3p, while concurrently increasing Suv39H1 expression^37^. Suv39H1, a histone methyltransferase, is implicated in promoting malignant growth and oncogenesis across various solid and hematological malignancies^38^.

Our study acknowledges certain limitations. Firstly, the sample size was relatively small, potentially restricting the generalizability of our findings. Secondly, the follow-up duration was insufficient to establish a correlation between the expression of these markers and patient survival outcomes. Finally, a comparison between serum and tissue expression levels of these markers was not performed, which could have provided additional insights into their clinical relevance.

## Conclusions and recommendations

Circulating levels of HOTAIR and miR-130a hold promise as non-invasive biomarkers for predicting the pathological features of breast cancer patients. HOTAIR, an oncogenic lncRNA, promotes tumor burden and metastasis, while miR-130a, a tumor suppressor miRNA, exhibits downregulation with advanced stage and metastasis of breast cancer. Further investigations are warranted to better elucidate the interaction between HOTAIR and miR-130a and determine their potential utility in early relapse prediction. Long-term follow-up studies are recommended to delineate the role of HOTAIR in breast cancer recurrence.

### Supplementary Information

Supplementary Information.

## Data Availability

The datasets used and/or analyzed during the current study are available from the corresponding author on reasonable request.
