# Author Correction: Circulating ESR1, long non-coding RNA HOTAIR and microRNA-130a gene expression as biomarkers for breast cancer stage and metastasis

**DOI:** 10.1038/s41598-024-52878-8

**Published:** 2024-02-02

**Authors:** Noura R. Abdel-hamid, Eman A. Mohammed, Eman A. Toraih, Mahmoud M. Kamel, Ahmed Samir Abdelhafiz, Fouad M. Badr

**Affiliations:** 1 Genetics Unit, Department of Histology and Cell Biology, Faculty of Medicine, Suez Canal University, Ismailia, Egypt; 2 Division of Endocrine and Oncologic Surgery, Department of Surgery, School of Medicine, Tulane University, New Orleans, LA 70112 USA; 3 Department of Clinical Pathology, National Cancer Institute, Cairo University, Kasr Al-Aini Street, From El-Khalig Square, Cairo, 11796 Egypt; 4 Baheya Centre for Early Detection and Treatment of Breast Cancer, Giza, Egypt

Correction to: *Scientific Reports* 10.1038/s41598-023-50007-5, published online 19 December 2023

The original version of this Article omitted an affiliation for Mahmoud M. Kamel. The correct affiliations are listed below.

Department of Clinical Pathology, National Cancer Institute, Cairo University, Kasr Al-Aini Street, From El-Khalig Square, Cairo, 11796, Egypt.

Baheya Centre for Early Detection and Treatment of Breast Cancer, Giza, Egypt.

The original Article has been corrected.

