# Supplementary material for: Circulating ESR1, long non-coding RNA HOTAIR and microRNA-130a gene expression as biomarkers for breast cancer stage and metastasis
Source: Sci Rep. 2023 Dec 19;13:22654. doi: 10.1038/s41598-023-50007-5 (PMC10730703; doi:10.1038/s41598-023-50007-5)
Supplement: Supplementary file 1 — Supplementary Information. [file 41598_2023_50007_MOESM1_ESM.docx]

**Supplementary files**

**Supplementary table 1: Sequences of primers used for gene amplification.**

| **HOTAIR** | - Forward primer sequence (5׳ to 3׳) GGTAGAAAAAGCAACCACGAAGC - Reverse primer sequence (3׳ to 5׳) ACATAAACCTCTGTCTGTGAGTGCC |
| --- | --- |
| **ESR1** | - Forward primer sequence (5׳ to 3׳) TCCTCATCCTCTCCCACATC - Reverse primer sequence (3׳ to 5׳) AGTGGCTTTGGTCCGTCTC |
| **GABDH** | - Forward primer sequence (5׳ to 3׳) CCCCGCTACTCCTCCTCCTAAG - Reverse primer sequence (3׳ to 5׳) TCCACGACCAGTTGTCCATTCC |

| **Supplementary table 2: Thermal cycling conditions of qRT-PCR for miRNA-130a and TBP**   \| **Stage** \| \| **Temperature (°C)** \| **Time (mm:ss)** \| \| --- \| --- \| --- \| --- \| \| Polymerase activation \| \| 95 \| 10:00 \| \| Cycle  (40 cycle) \| Denaturation \| 95 \| 00.15 \| \| Annealing/extension \| 60 \| 1:00 \|   **Supplementary table 3: Thermal cycling conditions of qRT-PCR for ESR1, HOTAIR and GABDH**   \| **Stage** \| \| **Temperature (°C)** \| **Time (mm:ss)** \| \| --- \| --- \| --- \| --- \| \| Initial denaturation and Polymerase activation \| \| 95 \| 2:00 \| \| Cycle  (45 cycle) \| Denaturation \| 95 \| 00.15 \| \| Annealing/extension \| 60-65* \| 1:00ǂ \|   **Supplementary table 4. Reproductive and personal characteristics of breast cancer patients (n=45).** | | |
| --- | --- | --- | --- | --- | --- | --- | --- | --- | --- | --- | --- | --- | --- | --- | --- | --- | --- | --- | --- | --- | --- | --- | --- | --- | --- | --- | --- | --- | --- | --- | --- | --- |
| **Variables** | **Frequency** | **Percentage** |
| **Age at menarche (y)** | 25  20 | 55.6%  44.4% |
| ˂12 |  |  |
| ≥12 |  |  |
| **Marital status** | 2  43 | 4.4%  95.6% |
| Single |  |  |
| Married |  |  |
| **Parity** | 2  43 | 4.4%  95.6% |
| Nullipara |  |  |
| Parous |  |  |
| **Age at first pregnancy (y)** |  |  |
| ˂30 | 34 | 79.1% |
| ≥30 | 9 | 20.9% |
| **History of breast feeding** |  |  |
| Yes | 40 | 88.9% |
| No | 5 | 11.1% |
| **Menopausal status** |  |  |
| Premenopausal | 23 | 51.1% |
| Postmenopausal | 22 | 48.9% |
| **Age at menopause (y)** |  |  |
| ˂55 | 13 | 28.9% |
| ≥55 | 9 | 20% |
| **Contraceptive use** |  |  |
| Yes | 32 | 71.1% |
| No | 13 | 28.9% |
| **History of benign breast disease** | 11 | 24.4% |
| **Family history of cancer**  Breast cancer  Others (Liver) | 4  1 | 8.8%  2.2% |
|  | | |

**Supplementary figure 1.** Receptor status of breast cancer patients (n=45). ER, estrogen receptor; PR, progesterone receptor.

**Supplemntary figure 2.** Intrinsic molecular class of breast cancer patients (n=45). Basal-like (triple negative), ER^-^, PR^-^, HER2^-^; Luminal A, ER^+^, PR^+^, HER2^-^; Luminal B, ER^+^, PR^+/-^, HER2^+^; HER2^+^ subset, ER^-^, PR^-^, HER2^+^.

Values are shown median (1st -3rd quartiles). Mann-Whitney and Kruskal Wallis tests were used. LN; Lymph node, LV; Lymphovascular and NPI; Nottingham Prognostic Index.

**
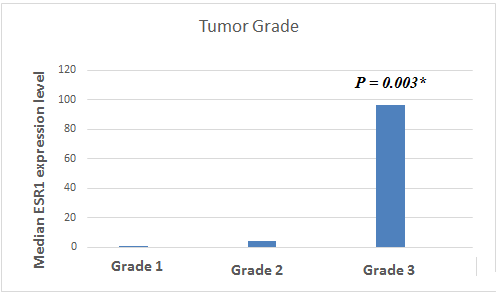
**

**Supplemntary figure 3. Association between ESR1 expression and Tumor grade (n=45).**


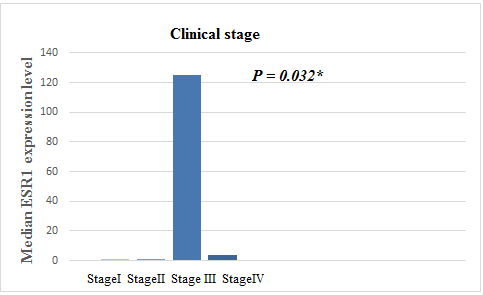


**Supplemntary figure 4. Association between ESR1 expression and Clinical stage (n=45).**

**
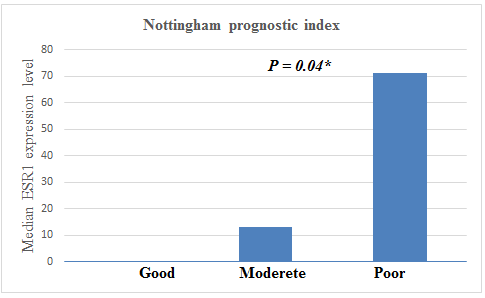
**

**Supplemntary figure 5. Association between ESR1 expression and Nottingham Prognostic Index.**
